# Supplementary material for: Impact of Dietary Patterns on Skeletal Health: A Systematic Review and Meta-Analysis of Bone Mineral Density, Fracture, Bone Turnover Markers, and Nutritional Status
Source: Nutrients. 2025 Dec 9;17(24):3845. doi: 10.3390/nu17243845 (PMC12735435; doi:10.3390/nu17243845)
Supplement: Supplementary file 1 [file nutrients-17-03845-s001.zip › nutrients-3987767-supplementary.pdf]

Supplementary Table S1. Methodological Quality of Included Non-Randomized Studies Based on JBI Checklist

Study (Year)	Study Design	Q1	Q2	Q3	Q4	Q5	Q6	Q7	Q8	Q9	Overall Risk
Antonio et al. (2022) [55]	Cross-sectional	Yes	Yes	Yes	Yes	No	–	Yes	–	Yes	Moderate
Benetou et al. (2018) [47]	Cohort	Yes	Yes	Yes	Yes	Yes	Yes	Yes	Yes	Yes	Low
Benetou et al. (2013) [48]	Cohort	Yes	Yes	Yes	Yes	Yes	Yes	Yes	Yes	Yes	Low
Byberg et al. (2016) [49]	Cohort	Yes	Yes	Yes	Yes	Yes	Yes	Yes	Yes	Yes	Low
Cervo et al. (2021) [27]	Cohort	Yes	Yes	Yes	Yes	Yes	Yes	Yes	Yes	Yes	Low
Chen et al. (2016) [56]	Cross-sectional	Yes	Yes	Yes	Yes	No	–	Yes	–	Yes	Moderate
Erkkilä et al. (2017) [50]	Cohort	Yes	Yes	Yes	Yes	Yes	Yes	Yes	Yes	Yes	Low
Féart et al. (2013) [51]	Cohort	Yes	Yes	Yes	Yes	Yes	Yes	Yes	Yes	Yes	Low
Fung et al. (2018) [52]	Cohort	Yes	Yes	Yes	Yes	Yes	Yes	Yes	Yes	Yes	Low
Haring et al. (2016) [53]	Cohort	Yes	Yes	Yes	Yes	Yes	Yes	Yes	Yes	Yes	Low
Lee et al. (2020) [57]	Cross-sectional	Yes	Yes	Yes	Yes	No	–	Yes	–	Yes	Moderate
Mitchell et al. (2021) [54]	Cohort	Yes	Yes	Yes	Yes	Yes	Yes	Yes	Yes	Yes	Low
Moradi et al. (2018) [58]	Cross-sectional	Yes	Yes	Yes	Yes	No	–	Yes	–	Yes	Moderate
Pérez-Rey et al. (2019) [59]	Cross-sectional	Yes	Yes	Yes	Yes	No	–	Yes	–	Yes	Moderate
Warensjö et al. (2021) [61]	Cohort	Yes	Yes	Yes	Yes	Yes	Yes	Yes	Yes	Yes	Low
Zeng et al. (2014) [60]	Case–control	Yes	Yes	Yes	Yes	Yes	Yes	Yes	–	Yes	Low

Note: "–" indicates item not applicable for that study design. Based on JBI Critical Appraisal Checklists (Joanna Briggs Institute, 2020).

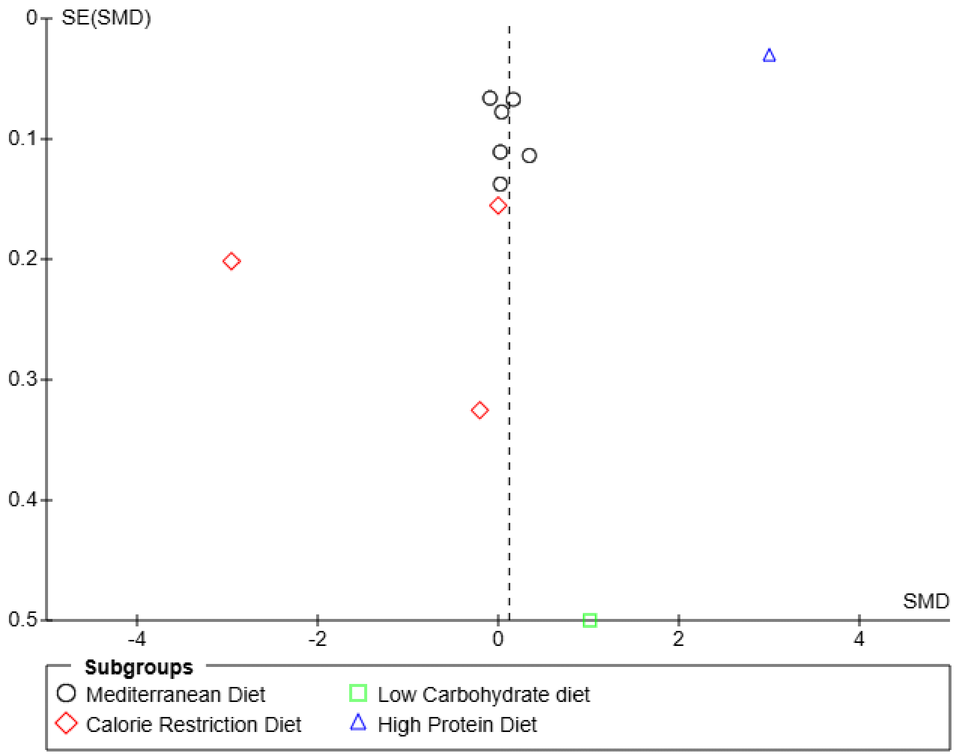

Figure S1: The funnel plot for femoral neck BMD revealed a largely symmetrical distribution of studies around the pooled effect size. The points were dispersed evenly across both sides of the mean line, indicating that small-study effects or publication bias were unlikely for this outcome. A few studies with larger standard errors appeared at the bottom of the plot, consistent with the expected random scatter of smaller trials. Overall, the pattern suggests low risk of publication bias for femoral neck BMD.

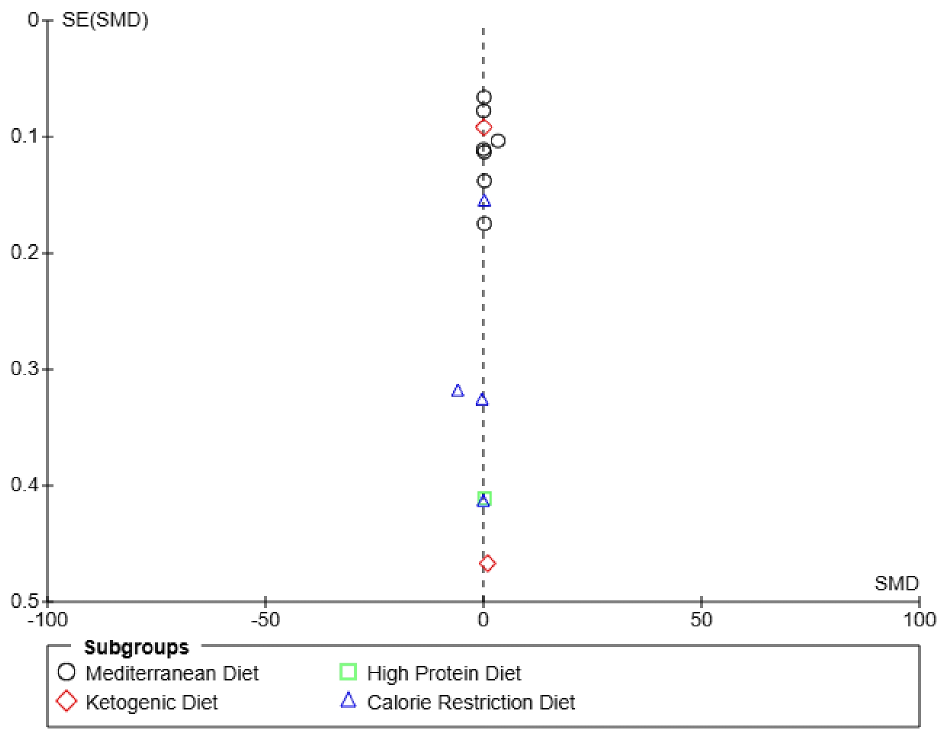

Figure S2: The funnel plot for lumbar spine BMD demonstrated slight asymmetry, with a minor clustering of smaller studies on one side of the mean effect. This could suggest a potential small-study effect or selective publication tendency, possibly favoring studies showing beneficial dietary impacts on BMD. However, the asymmetry was modest, and given the heterogeneity of included study designs and sample sizes, this pattern may also reflect true clinical diversity rather than publication bias. Therefore, the risk of publication bias for this outcome is considered moderate.

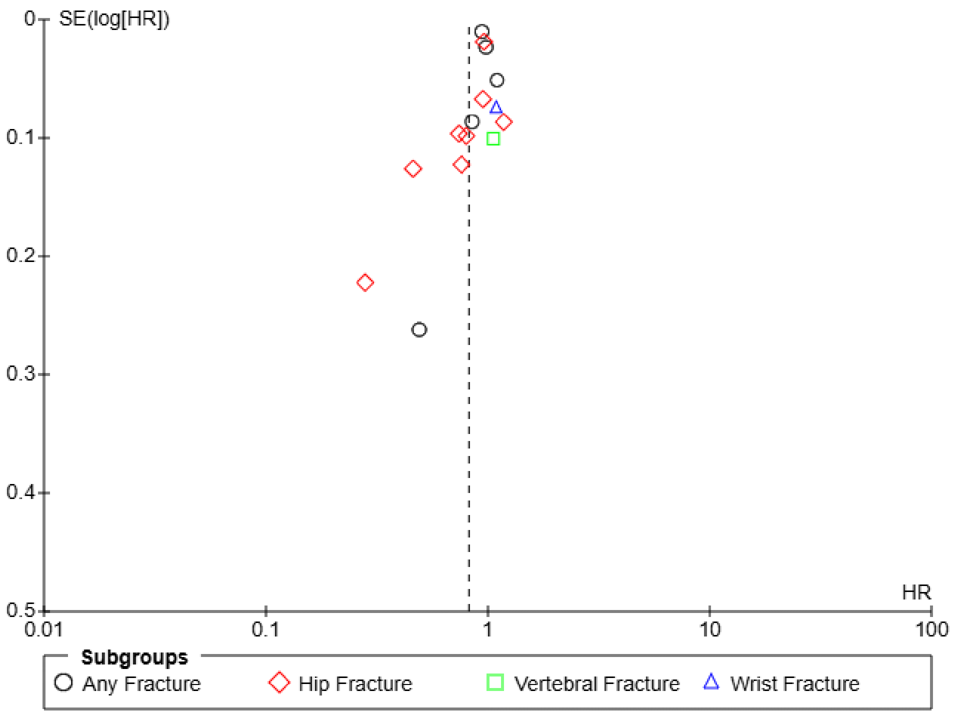

Figure S3: The funnel plot for fracture risk showed a relatively symmetrical distribution with most studies aligned closely around the pooled estimate and only slight dispersion among smaller studies. There was no marked evidence of asymmetry, suggesting a low publication bias. The larger cohort studies appeared near the top of the plot, reflecting high precision, and the absence of extreme outliers supports the robustness of the pooled estimate.
